# The Effect of Topical Oxygen Therapy in Horses Affected with Mycosis of the Guttural Pouch: An Experimental Pilot Study and a Case Series

**DOI:** 10.3390/ani11113329

**Published:** 2021-11-22

**Authors:** Olivier M. Lepage, Paola Di Francesco, Nicolas Moulin, Monika Gangl, Gaëtan Texier, Joffrey Marchi, Jean-Luc Cadoré

**Affiliations:** 1 Centre for Equine Health, Ecole Nationale Vétérinaire de Lyon, VetAgro Sup, University of Lyon, F-69280 Marcy l’Etoile, France; paoladf91@gmail.com (P.D.F.); nmoulindvm@gmail.com (N.M.); monika.gangl@vetagro-sup.fr (M.G.); jean-luc.cadore@vetagro-sup.fr (J.-L.C.); 2 French Armed Forces Center for Epidemiology and Public Health, F-13568 Marseille, France; gaetex1@gmail.com (G.T.); joffrey.marchi@gmail.com (J.M.); 3 UMR 912/SESSTIM-INSERM/IRD, Faculty of Medicine, F-13385 Marseille, France

**Keywords:** guttural pouch, mycosis, topical oxygen therapy, horse, dysphagia, transarterial coil embolization, inflammation

## Abstract

**Simple Summary:**

Resolution of macroscopic inflammatory lesions in case of guttural pouch mycosis (GPM) in horses is highly variable, and resolution of neurological disorders is inconstant and challenging. We hypothesized that topical oxygen therapy (TOT) would safely modulate GPM by reversing the course of the disease. The objective of a phase-1 pilot study was to validate the safety and feasibility of TOT in horses inoculated with *Aspergillus fumigatus*. The objective of a phase-2 clinical study was to report its effect in horses with a naturally occurring GPM. After more than two TOT administrations, inflammatory lesions decreased faster in size in the treated GP. In phase 2, partial or total recovery of neurological disorders (2/4 laryngeal hemiparesis, 3/5 dysphagia, 1/2 dorsal displacement of the soft palate, 1/1 Horner’s syndrome) was recorded. TOT can be recommended in two clinical presentations. If no history of epistaxis exists, and if lesions are not overlying major arteries, TOT alone can be instituted. In the case of a patient at risk of epistaxis, a multimodal approach with a transarterial coil embolization (TACE) procedure should be proposed. In both situations, TOT administration four times a day at 15 L/min for one to two weeks are probably sufficient to reverse the course of the disease.

**Abstract:**

**Background:** The management of bleeding originating from the guttural pouch (GP) has a high success rate, but the resolution of the macroscopic inflammatory lesions in the case of mycosis (GPM) is highly variable; the resolution of neurological disorders is inconstant and challenging. **Objectives:** Our aim was to test the feasibility and safety of topical oxygen therapy (TOT) in horses after induction of GPM and in cases with naturally occurring disease. **Study design:** This study was an in vivo experimental and retrospective two-phase study. **Methods:** During phase 1, the pilot study, both GPs were inoculated with *Aspergillus fumigatus*. One GP was randomly assigned to receive one to four TOT 30 min sessions with 100% medical oxygen at 9 L/min. Follow-up endoscopic images were assessed for scoring macroscopic inflammatory lesions of the pharynx and both GPs. In phase 2, the clinical study, TOT was administered for 45 to 60 min at 15 L/min in six horses presenting with GPM. **Results:** In phase 1, TOT administration was easy to perform in the standing horse with no adverse effects. After more than two administrations, macroscopic inflammatory lesions decreased more quickly in size in the treated GP. In phase 2, horses were treated with TOT only (*n* = 1) or combined with a transarterial coil embolization (TACE) procedure (*n* = 5). After TOT and discharge from the hospital, nasal discharge resolved in three horses, and improvement was noted in the fourth one. Between days 2 and 10 after admission, upper respiratory tract endoscopy (URTE) indicated size reduction and alteration in the appearance of all the macroscopic inflammatory lesions. The partial or total recovery of neurological disorders (2/4 laryngeal hemiparesis, 3/5 dysphagia, 1/2 dorsal displacement of the soft palate (DDSP), and 1/1 Horner’s syndrome) was recorded. **Main limitations:** In phase 1, the small number of horses did not allow for statistically significant conclusions; in phase 2, clinical signs at admission varied between horses, which made comparison difficult. **Conclusions:** In adult horses, TOT alone or in combination with TACE is feasible and safe with a propensity to reverse the course and the progression of inflammatory lesions without additional local or systemic treatment.

## 1. Introduction

Guttural pouch mycosis (GPM) is a rare but sometimes life-threatening disease [1]. The signs of GPM are nasal discharge, neurological disorders, and epistaxis. With endoscopy, the size, location, and appearance of macroscopic inflammatory lesions of the mucosa are found to be variable and not necessarily related to the severity of the clinical signs. The plaques present with different thicknesses and colors, sometimes covered with a diphtheritic membrane and brilliant white mycelium. Mycological culture often highlights *Aspergilli*, ubiquitous throughout the environment, found in organic debris, and considered to be involved in the pathogenesis of GPM [2]. The pathological appearance of invasive *Aspergilli* shows infarcted tissue invaded by fungal hyphae [3] found in the arterial walls [2] or nerves [4]. The management of bleeding is effective with transarterial coil embolization (TACE) [5], but if neurological disorders are present, they only resolve in approximately 50% of patients [6]. This poor outcome explains why there is a need to use additional treatments to arterial occlusions, such as topical antifungal therapy [7], or a means of modifying the GP environment by performing a salpingopharyngostomy [8]. Spontaneous regression of GPM has been observed experimentally [9] and in clinical cases [5], but the pathogenic mechanism remains unknown.

Even though *Aspergillus* species are highly aerobic and found in almost all oxygen environments, they are also capable of growth at low oxygen tension, which may have implications for their pathogenicity, possibly by affecting the secretion of extracellular virulence factors [10]. Paradoxically, increased oxygen levels at the site of infection reduce fungal biofilm proliferation and the deleterious effect of *Aspergillus* [11]. The effect is transient and fungistatic, with *Aspergillus* metabolic activity rebounding within six hours of oxygen therapy being removed. Hypoxia or low oxygen levels at an *Aspergillus* infection site also impairs the killing capacity and phagocytosis of neutrophils and macrophages [12,13]. Oxygen therapy is beneficial in the treatment of human conditions such as invasive pulmonary aspergillosis [11], diabetic foot ulcers [14], and chronic soft tissue infections [15]. It is a potent antifungal that improves polymorphonuclear function [16,17]. It is also toxic to anaerobic bacteria [18], and, in the case of clostridial myonecrosis, it can stop the production of the alpha toxin [19]. Two major methods are used to deliver oxygen to target tissue: hyperbaric oxygen and topical oxygen therapy (TOT) [20,21]. Topical oxygen therapy is the medical use of 100% oxygen in a normobaric situation with the ability of oxygen to penetrate the tissue directly from the surface and not from the disrupted vasculature.

Given the positive experience with oxygen therapy in the resolution of human fungal disease [16] and its reported use in horses [22,23], we conducted a two-phase study to assess TOT for the treatment of GPM in horses [24]. We hypothesized that TOT would safely modulate guttural pouch mycosis in horses by reversing the course of the disease. The objective of the phase-1 pilot study was to validate the safety and feasibility of TOT in horses inoculated with *Aspergillus fumigatus*. The objective of the phase-2 clinical study was to determine the effect of TOT in horses with naturally occurring GPM.

## 2. Materials and Methods

The phase-1 part of the study was reviewed, approved, and performed according to the guidelines of the Animal Ethics Committee of VetAgro Sup under No. 1605; and reviewed, approved, and performed according to the guidelines of the French Animal Ethics Committee under No. AP AFIS#4464-20 160 11415186986-018. Animals included in the phase-2 clinical part of the study were treated following the owners’ written consent.

## 3. Phase 1 Pilot Study

**Figure 1:**
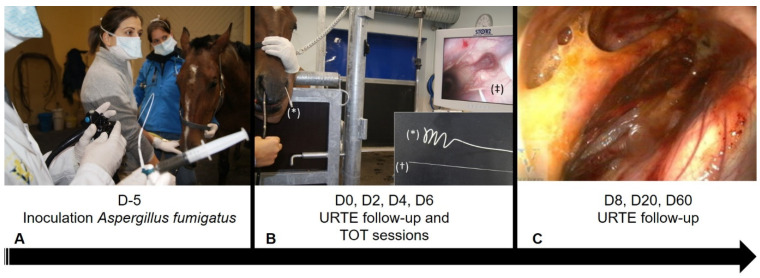
Experimental design and timing of the phase 1 pilot study: (**A**) five days before the first TOT (topical oxygen therapy) session, all GPs (guttural pouch) were inoculated with *Aspergillus fumigatus*; (**B**) after URTE (upper respiratory tract endoscopy) assessment, a specific catheter was placed under endoscopic guidance into the GP for oxygen administration. The catheter mounted on a metallic guidewire (†) was advanced on the ventral nasal meatus into the GP (‡). Luer lock and spiraled multiple fenestrated ends of the catheter are visualized (*); (**C**) follow-up URTEs were performed until day 60.

Eight adult French Trotters (three mares and five geldings) free of any clinically detectable disorder of the pharynx or GPs at upper respiratory tract endoscopy (URTE) were included in the study. They were kept by pairs in small paddocks under constant conditions. Five days (Figure 1A) prior to the first TOT session (D-5), horses were sedated with 0.01 mg/kg detomidine (Domosedan, Pfizer, Paris, France) and 0.04 mg/kg of butorphanol tartrate (Torbugesic, Fort Dodge, Tours, France)

**Figure 2:**
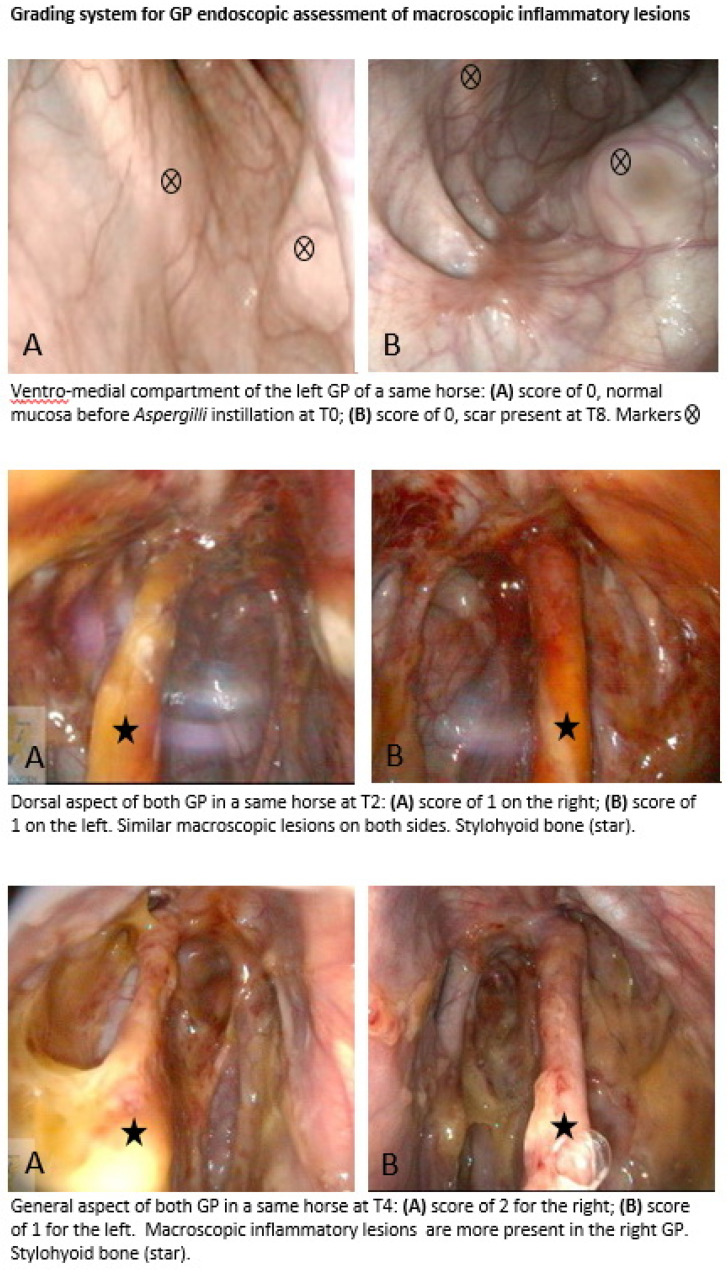
Endoscopic grading system of macroscopic inflammatory lesions present in the GP.

A URTE was performed to evaluate for any abnormalities of the nasopharynx and both GPs before inoculation with the strain *Aspergillus fumigatus* CBS 144.89 (EA 7380, Dynamyc, EnvA, Maison-Alfort, France). In each pouch, approximately 9 × 10^5^ conidia in a 15 mL final volume of sterile physiologic saline were inoculated by an endoscopy-guided technique [9]. Individuals were randomly assigned by pair to one of four treatment groups: Group 1 (one TOT session on D0); Group 2 (two TOT sessions 48 h apart on D0 and D2); Group 3 (three TOT sessions 48 h apart on D0, D2, and D4); Group 4 (four TOT sessions 48 h apart on D0, D2, D4, and D6). A simple randomization method was used to allocate which GP was treated. On the first day of treatment (D0), horses were sedated intravenously with 0.01 mg/kg detomidine (Domosedan, Pfizer, Paris, France) and 0.04 mg/kg of butorphanol tartrate (Torbugesic, Fort Dodge, Tours, France). Before each treatment, the endoscope was placed in the contralateral nasal passage of the GP to be treated to enable the observation of the placement of a specific, purpose-made 8 Fr, 100 cm long silicone catheter, COOK, Eight Mile Plains, Australia, with multiple fenestrations on its spiraled end (Figure 1B). The outside part of the catheter was secured to the halter and connected via its Luer lock extremity to an extender adapted to a 5 L bottle of medicinal oxygen at 200 bars. Oxygen was administered at a rate of 9 L/min for 30 min per session. Horses remained standing in stocks during the entire procedure with the head attached on both sides of the front gate. The GP catheter was removed at the end of each treatment session. Follow-up URTE (Figure 1C) was performed eight times (T1–T8): before *Aspergillus* inoculation (T1 on D-5), just before the first TOT session on D0 (T2), and at days D2 (T3), D4 (T4), D6 (T5), D8 (T6), D20 (T7), and D60 (T8). All data were recorded with a PC AIDA system, Karl Storz, Guyancourt, France. Each URTE follow-up period included images captured of nine locations: one of the pharynx including both pharyngeal GP orifices; the epiglottis and arytenoids for each GP; one general view; one image centered on the proximal third of the stylohyoid bone and the roof of the GP; one image of the ventral lateral compartment; one of the ventral medial compartment. Images were always captured prior to oxygen administration if a TOT session was planned. For each of the eight URTE monitoring periods (T1–T8), all nine images were included in a standardized PowerPoint slide show presented to six evaluators. These included one senior surgeon and one intern directly involved with the study, and four equine residents (two surgery, one medicine, and one reproduction) from the same institution, not involved in the study. Evaluators were seated apart in the same projection room during the assessment to avoid any interaction. Evaluators, blinded to which pouch received TOT, assessed the images for the presence of discharge at the GP pharyngeal orifices and for the presence of macroscopic inflammatory lesions in the GPs. Macroscopic inflammatory lesions consisted of (1) hyperemic mucosa; (2) orange exudate; (3) plaques of adherent, dense, and beige to orange material; (4) grey pseudo-membrane; (5) brilliant white mycelia. A score of 0, 1, or 2 was assigned to each GP in comparison with the contralateral GP in the same horse during the same monitoring period (Figure 2). A score of 0 means no anomalies or presence of scar tissue without any other macroscopic inflammatory lesions. A score of 1 means the presence of macroscopic inflammatory lesions equally in both GP. A score of 2 indicates the presence of macroscopic lesions at a higher proportion than the contralateral GP.

## 4. Statistical Analysis

Statistical analyses were performed for the phase-1 pilot study. To control for the possible effect of the evaluator on image evaluation (when scoring for the presence of macroscopic inflammatory lesions), a measure of agreement was calculated between the six evaluators. Due to a multiple judgment problem with ordinal data with replicates, the agreement was tested using Krippendorff’s alpha coefficient. A Krippendorff’s alpha of <0 indicates a poor agreement, 0 to 0.2 indicates a slight agreement, 0.21 to 0.40 indicates a fair agreement, 0.41 to 0.60 denotes a moderate agreement, 0.61 to 0.80 indicates a substantial agreement, and between 0.81 to 1 indicates near-perfect agreement. To assess the difference between GP treated and untreated, the Kolmogorov–Smirnov test was used. All statistical analyses were conducted with statistical software R Core Team (R: A language and environment for statistical computing. Foundation for Statistical Computing, Vienna, Austria. URL (https://www.R-project.org/, accessed on 20 December 2016) version 3.3.0, 2016).

## 5. Phase-2 Clinical Study

After the completion of the phase-1 pilot study, horses presenting with GPM were included in the phase-2 clinical study (Table 1). A commercial 8 Fr 135 cm long GP polyurethane catheter (MILA International Inc., Florence, SC, USA) placed under endoscopic guidance was used for TOT administration. It was left in place as long as possible and replaced only if it fell out of the GP. Since the pilot study showed no adverse effects of TOT after a 30 min session, time was increased from 45 to 60 min at 15 L/min in the clinical study. If arterial occlusion was necessary, a TACE procedure, as previously described [25], using embolization coils (Nester^®^) (COOK medical, Charenton-le-Pont, France) was performed. Dysphagia was described by a grading system out of 4: grade 1, cough (intermittent, not necessarily related to meals) and no nasal discharge; grade 2, cough (related to meals) and no nasal discharge; grade 3, cough (related to meals) and alimentary nasal discharge; grade 4, cough (related to meals), alimentary nasal discharge, and weight loss. The Havemeyer endoscopic laryngeal grading system was used to describe the laryngeal function [26].

## 6. Horse 1

### 6.1. Case History

Horse 1 had a history of a 2-month duration of dysphagia with nasal discharge containing food material and weight loss. No history of epistaxis was reported.

### 6.2. Diagnosis

At admission, grade 4 dysphagia was present, and a URTE without sedation revealed food material in the nasopharynx and upper trachea, permanent DDSP, and complete immobility of the left arytenoid cartilage categorized as a grade IV laryngeal hemiparesis. Examination of the left GP revealed fungal plaques with focal white mycelia on the stylohyoid bone, the internal carotid artery (ICA), glossopharyngeal, and hypoglossal nerves. The right GP was normal. Blood biochemical and hematological analysis revealed no changes except for mild lymphopenia (1.32 × 10^9^ cells/L; reference range (rr): 1.50–7.70 × 10^9^ cells/L).

### 6.3. Treatment

**Figure 3:**
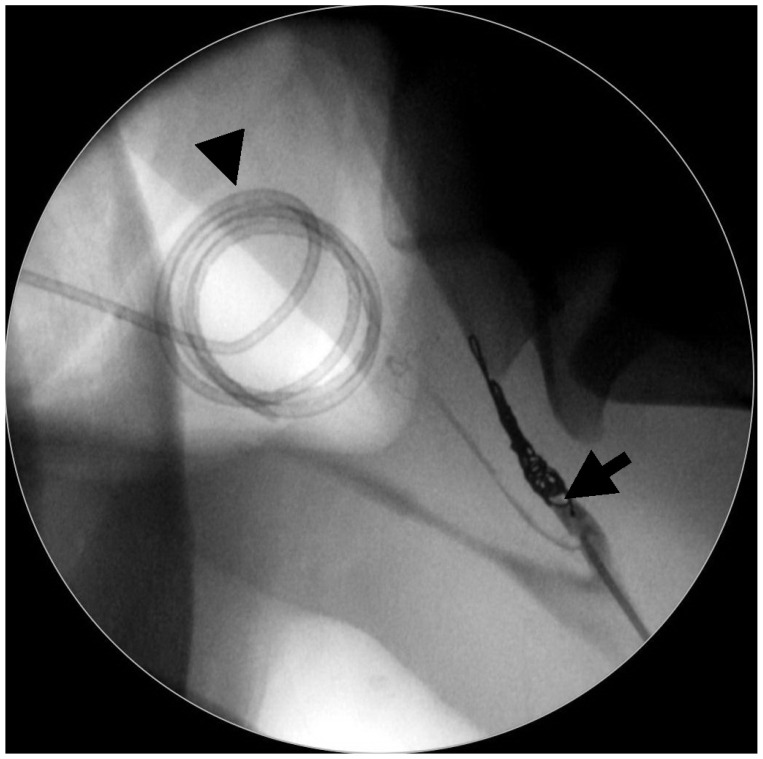
Fluoroscopic image obtained during general anesthesia of Horse 1. A TACE (transarterial coil embolization) procedure of the left ICA (internal carotid artery) (arrow) was performed simultaneously with a TOT (topical oxygen therapy) session. Note the spiraled catheter for oxygen administration sitting into the left GP (guttural pouch) (arrowhead).

Before inducing general anesthesia to perform a TACE of the left ICA, flunixin meglumine (1.1 mg/kg i.v.; Antalzen, Laboratorios Calier S.A., Barcelona, Spain) and perioperative antibiotic therapy using trimethoprim–sulfonamide (25 mg/kg i.v.; Borgal 24%, Virbac, Carros, France) were administered. Under general anesthesia, a first 30 min TOT session was performed concomitantly with a TACE procedure (Figure 3). Post-operative antibiotics and pain relief were provided for 9 days using twice-daily trimethoprim–sulfonamide (25 mg/kg i.v.; Avemix, Vetoquinol S.A., Lure, France) and twice-daily phenylbutazone (2.2 mg/kg p.o.; Equipalazone, Dechra veterinary products S.A.S, Montigny-le-Bretonneux, France). TOT sessions were performed twice daily for five days. Immediately after surgery, the horse was still coughing during meals, but there was no problem when drinking water. Two days postsurgery, URTE revealed permanent DDSP, complete immobility of the left arytenoid cartilage, and a non-pulsatile left ICA. Macroscopic modification, such as the absence of white mycelia and liquefaction of other macroscopic lesions, was observed. Seven days later, both nostrils were clean and the horse was able to eat slowly, showing fewer coughing episodes (grade 3 dysphagia). URTE revealed a permanent DDSP, a grade III-2 laryngeal hemiparesis, and a homogenous reduction in the size of all macroscopic inflammatory lesions with fungal plaques replaced by melting beige-orange lesions. The horse was discharged from the hospital the same day with a five-day prescription of trimethoprim–sulfonamide (25 mg/kg per os; Avemix, Vetoquinol S.A., Lure, France) and twice-daily phenylbutazone (2.2 mg/kg per os; Equipalazone, Dechra veterinary products S.A.S, Montigny-le-Bretonneux, France).

### 6.4. Outcome

Follow-up 3 months later was obtained by telephone interview with the owner and images sent by mail. This recreational horse was recovering weight and had only a few and irregular episodes of coughing associated with eating (grade 2 dysphagia).

## 7. Horse 2

### 7.1. Case History

Horse 2 had a history of bilateral mucopurulent nasal discharge, with food material, of 3 weeks’ duration. Initially, she had been unsuccessfully treated with a one-week course of inhalation therapy (drugs unknown).

### 7.2. Diagnosis

**Figure 4:**
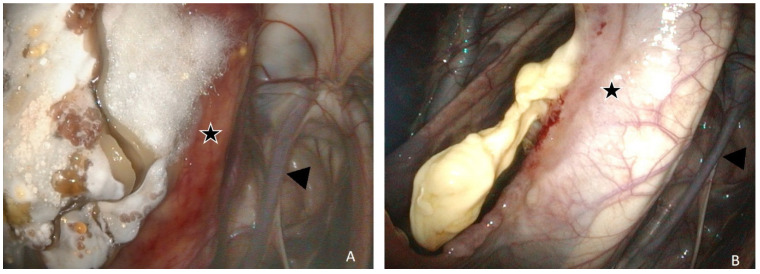
Endoscopic evaluation of Horse 2. Left GP (guttural pouch) presented with bilateral mycosis and treated only with TOT (topical oxygen therapy): (**A**) at admission, a thick granulomatous plaque covered with white brilliant mycelia is located on the median septum (star); (**B**) after 27 days’ of TOT, the absence of mycotic lesions with the exception of a small amount of necrotic tissue in the scarring of a large septum fistula. Arrowhead, left ICA (internal carotid artery).

A grade 3 dysphagia was present at admission. A URTE at rest revealed a grade II left laryngeal hemiparesis and yellowish mucous discharge from both GP pharyngeal orifices. Multiple granulomas and thick dark-beige plaques covered with multiple diphtheritic lesions with brilliant white mycelia were present in the medial compartment of both GPs, adjacent to the right ICA and involving the median septum (Figure 4). *Aspergillus nidulans* was later isolated from an endoscopic biopsy of these lesions.

### 7.3. Treatment

As there was no history of epistaxis, and fungal lesions were about 2 mm from the right ICA and about 2 cm from the left ICA, no TACE procedure was proposed. Bilateral TOT sessions were performed twice daily. No antibiotics or NSAIDs (Nonsteroidal anti-inflammatory drugs) were administered during hospitalization. After two days of TOT sessions, URTE revealed liquefaction or a melting-like appearance of the lesions, a sharp decrease in the size of diphtheritic plaques, the absence of white mycelia, and the presence of an orange exudate type of fluid covering abnormal and normal respiratory mucous membranes. Follow-up URTE after 17 days showed fistulization by necrosis of the GP septum and some macroscopic inflammatory lesions at this location. After 27 days, URTE revealed a resolution of the laryngeal hemiparesis at rest (grade I) and complete resolution of macroscopic inflammatory lesions with the exception of a small amount of necrotic tissue in the septum (Figure 4). The horse was discharged from the hospital the same day with no medication, no cough, and no nasal discharge.

### 7.4. Outcome

Four and six months after admission, the mare returned to training and won her first race.

## 8. Horse 3

### 8.1. Case History

Horse 3 had a history of recent bilateral epistaxis in the pasture, and the presence of clotted blood at the left pharyngeal GP orifice during a URTE by the referring veterinarian.

### 8.2. Diagnosis

URTE upon admission revealed a collapse of the left dorsal pharynx, a grade II left laryngeal hemiparesis, a clot filling the left GP, some dry blood on both nostrils, and an episode of coughing during the exam. To prevent the risk of further hemorrhage, the GPs were not examined. Blood biochemical and hematological analysis revealed mild anemia, hematocrit 21% (rr 30.0–47.0%), red cells 5.23 × 10^12^ cells/L (rr: 6.4–10.40 × 10^12^ cells/L).

### 8.3. Treatment

Treatment was initiated using procaine penicillin G (22.000 IU/kg i.m.; Depocilline, Intervet, Beaucouzé, France), gentamycin (6.6 mg/kg i.v.; G4, Virbac, Carros, France), and flunixin meglumine (1.1 mg/kg i.v.; Antalzen, Laboratorios Calier S.A., Barcelona, Spain) administered before inducing general anesthesia to perform a TACE procedure to occlude the left IC, external carotid (EC), and maxillary arteries. Post-operative antibiotics procaine penicillin G (22.000 IU/kg i.m. twice daily, Intervet, Beaucouzé, France) and gentamycin (6.6 mg/kg i.v. once daily, Virbac, Carros, France) were provided for 5 days, and flunixin meglumine (1.1 mg/kg i.v., Laboratorios Calier S.A., Barcelona, Spain) was administered once per day for 3 days. Four days after surgery, the clot had sufficiently drained out of the left GP to allow 45 to 60 min of TOT administration twice daily for 5 days. Grade 2 dysphagia was observed immediately after surgery and disappeared in the next 48 h. After three days of oxygen administration, it was possible to identify the left ICA as the source of hemorrhage. Multiple fungal lesions were present and seemed to melt along the entire surface of the styloid bone, the roof, and the middle part of both left GP compartments. When released from the hospital 10 days after admission, URTE revealed an absence of dorsal pharyngeal collapse, normal laryngeal function (grade I), and remnants of fungal lesions with a more liquefied aspect and tendency to drain to the GP floor.

### 8.4. Outcome

Within six months of discharge, the horse returned to normal endurance riding activities.

## 9. Horse 4

### 9.1. Case History

Horse 4 had a history of unilateral acute epistaxis at pasture. Before referring the animal to the hospital, at URTE, the veterinarian visualized blood coming from the right pharyngeal orifice and immediately performed an ipsilateral common carotid artery ligation. At admission to our referral hospital, URTE revealed a normal pharynx at the exception of clotting blood at the right pharyngeal orifice and into the ipsilateral GP without any signs of fungus or other infection. The left GP was normal. A TACE procedure was immediately performed under general anesthesia. During surgery, fluoroscopic guidance for the TACE procedure clearly demonstrated an aneurysm of the right ICA. Four days postsurgery, the horse was discharged from the hospital after a URTE showing few blood clots mainly on the floor of the GP, and no macroscopic inflammatory lesions of mycosis or bacterial infection. At home, the mare was back to her normal activity to the owners’ satisfaction. Two months after surgery, a routine URTE of the nasopharynx and both GPs by the referring veterinarian revealed a right non-pulsatile ICA and a significant right GPM with lesions mainly located on the roof and the entire medial compartment. The horse was referred for a second opinion and treatment to our hospital.

### 9.2. Diagnosis

At admission, the mare was clinically normal with no nasal discharge, no neurologic disorder, and no epistaxis. URTE confirmed a right GPM with diphtheritic lesions and brilliant white mycelia. The left GP was normal. Blood biochemical and hematological analysis revealed no abnormal changes.

### 9.3. Treatment

No treatment was administered with the exception of TOT sessions of the right GP three times a day for 15 days. After 5 days, URTE revealed melting-like lesions and size reduction in all lesions. Some of these were debrided with biopsy forceps, passed via the biopsy channel of the endoscope. At 14 days, only a small yellowish necrotic lesion, surrounded by a hyperemic scarring border, remained present, and the mare was discharged from the hospital.

### 9.4. Outcome

The mare returned immediately to her normal sports activity and remained normal six months later based on an interview with the owner.

## 10. Horse 5

### 10.1. Case History

Horse 5 presented with a one-week-duration, bilateral, mucopurulent, nasal discharge, with coughing and dysphagia. The owner noted a recent loss of condition of their horse. The referring veterinarian diagnosed with URTE a grade IV right laryngeal hemiplegia, the dorsal collapse of the pharynx, fluid accumulation around the epiglottis, food material in the nasopharynx and in the trachea, discharge from both GP orifices, and a bilateral GPM. Penicillin procaine (Depocilline, Intervet, Beaucouzé, France), gentamycin (G4, Virbac, Carros, France), metronidazole (Metrobactin, Dechra veterinary products S.A.S, Montigny-le-Bretonneux, France), flunixin meglumine (Antalzen, Laboratorios Calier S.A., Barcelona, Spain), and omeprazole (Pepticure, Audevard, Clichy, France) were immediately administered. Nebulization with a mix of gentamycin–dexadreson–balsaneb (Green Pex, Samoreau, France) once daily during two days of hospitalization was performed before referring the horse to our hospital for surgery and further treatments.

### 10.2. Diagnosis

**Figure 5:**
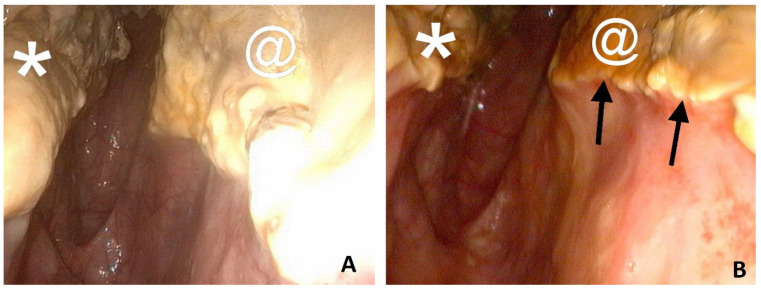
Endoscopic evaluation of Horse 5’s right GP (guttural pouch): (**A**) at admission before a TACE (transarterial coil embolization) procedure. Macroscopic inflammatory lesions are located in the medial ventral compartment involving the stylohyoid bone (*) and the median septum (@); (**B**) after 7 days of TOT (topical oxygen) and a TACE procedure, the volume occupied by the lesions located on the septum reduced and a scar line developed on the periphery (black arrows).

At admission, based on history and the clinical exam, grade 4 dysphagia and right ptosis of the upper eyelid compatible with Horner’s syndrome were observed. A URTE at rest, without sedation, revealed a grade IV right laryngeal hemiplegia, abnormally long periods of dorsal displacement of the soft palate (>15 s) not responding to stimulation with the endoscope, food material in the nasopharynx, and mucous discharge from both GP pharyngeal orifices, more notable on the right side. Examination of both GPs revealed thick beige plaques and granuloma-like structures covered sometimes with diphtheritic lesions and white mycelia throughout the entire right GP, preventing recognition of normal dorsal anatomical structures, with the exception of the maxillary artery and distal ECA. Similar lesions involving the median septum on the right GP (Figure 5A) were also present on the left GP but not adjacent to the ICA. All other physical examination findings were within normal limits. Blood biochemical and hematological analysis revealed no changes.

### 10.3. Treatment

A TACE procedure under general anesthesia for occlusion of the right internal and external carotid arteries was performed. A first 50 min TOT session was performed during the TACE procedure and continued for 15 days, four times daily. Due to the dysphagia and the high risk of aspiration pneumonia, postoperative antibiotics were administered twice daily trimethoprim–sulfonamide (25 mg/kg per os; Avemix, Vetoquinol S.A., Lure, France) for 5 days, metronidazole (15 mg/kg per rectum; Metrobactin, Dechra veterinary products S.A.S, Montigny-le-Bretonneux, France) three times daily for 3 days and flunixin meglumine (1.1 mg/kg i.v.; Antalzen, Laboratorios Calier S.A., Barcelona, Spain) twice daily for 4 days were administered.

Coughing associated with eating but not drinking was present immediately after surgery. The horse’s diet consisted of soaked mash and grazing periods. During the week following surgery, coughing with eating progressively decreased, and wet hay was introduced into the diet. As the risk of aspiration pneumonia appeared to be low, postoperative antibiotics and NSAIDs were stopped after 4 and 5 days, respectively. URTE two days after surgery revealed color modification and size reduction of the mycotic lesions in the right GP with a small hole perforating the septum between both GPs. On day 7, Horner’s syndrome was absent, and an important bilateral mucoexudative (orange-brown) nasal discharge was present. URTE revealed the absence of DDSP, exudative discharge from both pharyngeal orifices, a melting-like aspect of the mycotic lesions mixed with orange-brown exudate covering most of the GP respiratory mucosa, and a reduction in the size of the mycotic lesions. A scar line developed on the periphery of several lesions (Figure 5), some of which were debrided, in slices, with biopsy forceps passed via the biopsy channel of the endoscope. Detached necrosis slices were left on the floor of the GP. The hole in the medium septum was large enough to pass the endoscope from the right to the left GP. The GP catheter, being partially moved and damaged, was changed. Grain was introduced to the diet. On day 14 after admission, grade 2 dysphagia was still present with rare coughing episodes related to meals. Nasal secretions no longer contained food material and sharply declined. The GP catheter was removed, and the mare was discharged from the hospital.

### 10.4. Outcome

A follow-up 2 months later was obtained by telephone interview with the referring veterinarian and the URTE movie was captured under sedation and sent by mail. Nasal discharge was absent, the mare has not lost weight yet coughed sometimes and a grade III-2 right laryngeal hemiparesis was still present. At 4 months, the mare was pregnant, living normally in the pasture with other individuals.

## 11. Horse 6

### 11.1. Case History

Horse 6 was admitted with a history of 6 weeks of neck stiffness and right unilateral and later bilateral nasal discharge. No history of epistaxis was reported. The referring clinic made a diagnosis of right GPM, which was treated locally for 3 weeks with instillation of enilconazole (Imaveral^x^), miconazole (Daktarin^y^), and an iodine solution. Right guttural pouch instillation with miconazole (Daktarin^y^) and an iodine solution was continued for another 3 weeks. Due to a progressive worsening of the condition for 10 days with the appearance of coughing episodes, the pony was referred for a second opinion and treatment at our hospital.

### 11.2. Diagnosis

**Figure 6:**
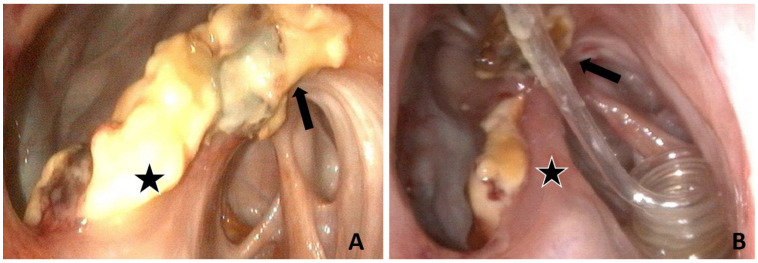
Endoscopic evaluation of Horse 6, right GP (guttural pouch): (**A**) at admission, macroscopic inflammatory lesions located on the stylohyoid bone (star) and the roof the medial compartment with regard to the ICA (internal carotid artery) (arrow); (**B**) after 9 days of TOT (topical oxygen therapy) and a TACE (transarterial coil embolization) procedure, the volume occupied by the macroscopic inflammatory lesion is reduced. The spiraled tip of the GP catheter is present in the medial compartment.

At admission, grade 2 dysphagia was present. URTE without sedation revealed a normal nasopharynx with the exception of secretions in the right GP orifice. The left GP was normal, but the right GP showed macroscopic inflammatory lesions located on the stylohyoid bone and the roof of the medial compartment covering the rostral extremity and the sigmoid flexure of the ICA (Figure 6). Blood biochemical and hematological analyses were within normal limits.

### 11.3. Treatment

Before inducing general anesthesia to perform a TACE of the right ICA, flunixin meglumine (1.1 mg/kg i.v.; Antalzen, Laboratorios Calier S.A., Barcelona, Spain) and perioperative procaine penicillin G (22.000 IU/kg i.m.; Depocilline, Intervet, Beaucouzé, France) were administered and continued for 24 h after surgery. Under general anesthesia, a first 45 min TOT session was performed concomitant with the TACE procedure and continued three times daily for 9 days. After this period, nasal discharge and coughing were resolved and URTE revealed a non-pulsatile right ICA with a reduction in the size of all macroscopic inflammatory lesions (Figure 6B). The horse was discharged from the hospital the same day.

### 11.4. Outcome

A follow-up 6 weeks later was obtained by a telephone interview with the owner. This pony was back to normal event training. Nasal discharge and coughing were not reported at any time during the convalescence.

## 12. Results

### 12.1. Phase-1 Pilot Study

All horses completed the study with no adverse effects due to TOT. Interevaluator agreement, assessed by the Krippendorff’s alpha coefficient, was fair (T3), substantial (T4, T6, and T7), or near perfect (T1, T2, T5, and T8) (Supplementary Item S1). Macroscopic inflammatory lesion assessment based on individual images included in a slide show showed a score of 0 at D-5 (T1) and D60 (T8) (Supplementary Item S2). At D60 (T8), various amounts of scar tissue were observed. At T2, the score was different from zero for all the horses, showing effective *Aspergillus fumigatus* inoculation. No statistical difference in the score between the treated and untreated GP was observed at T3 after one TOT session. From the second TOT (T4), the score allocated to the treated GP was consistently lower than the score of the nontreated GP in the same horse with a substantial (T4, T6, and T7) or near-perfect (T5 and T8) interevaluator agreement. A lower score was observed for the treated GP after three or four TOT administrations for all horses in Groups 3 and 4. Despite these observations, the differences observed were not significant (*p* = 0.09) (Supplementary Item S3).

### 12.2. Phase 2 Clinical Study

The main reasons for referral were epistaxis (*n* = 1), bilateral nasal discharge (*n* = 4), and dysphagia (*n* = 5). In three cases, the first TOT administration was performed under general anesthesia (H1, H5, and H6) during TACE. Two horses (H2 and H4) were treated only with TOT sessions, but Horse 4 had undergone a TACE procedure 2 months earlier to occlude an aneurysm of the ICA. The four remaining individuals (H1, H3, H5, and H6) underwent a TACE procedure, received perioperative antibiotics and NSAIDs including for the treatment of bacterial aspiration pneumonia. Oxygenotherapy was administered for 6 to 27 days, two to four times daily, at 15 L/min, for a period of 45 to 60 min through an indwelling catheter placed in each affected GP. In two horses (H4 and H5), partial and superficial removal of inflammatory lesions was performed once during URTE because major arteries were already occluded. When discharged from hospital, epistaxis had resolved (H3), mucopurulent discharge was absent in three horses (H1, H2, and H6), and a marked decrease was observed in the fourth one (H5). Five individuals (H1, H2, H3, H5, and H6) presented with one or more neurological disorders. Both horses with grade II laryngeal hemiparesis (H2, H3) recovered completely; two horses with grade IV (H1 and H5) improved to grade III-2. Improvement (H1 and H5) or absence of dysphagia (H2, H3, and H6) was observed in the five affected horses. Total recovery of DDSP was recorded in one of the two affected individuals (H5) and the only horse presenting with Horner’s syndrome (H5) recovered completely.

## 13. Discussion

The pathogenesis of guttural pouch mycosis remains a mystery in the horse [24]. For example, we do not find a compromised immune system to explain the increased virulence of *Aspergillus* as in human invasive pulmonary aspergillosis. In equids, GPM is most likely a multifactorial disease that depends on several different factors that contribute to its development such as the presence of aspergillus as well as bacteria and necrotic tissue. Even if we do not yet fully understand the etiopathology and the pathophysiology of GPM, advances have been achieved to prevent or treat the symptoms of the condition. Despite the very good results in surgically managing hemorrhage [25], we are still waiting for significant progress to help in the management of necrosis and bacterial and fungal growths within the complex microenvironment of the GP.

This two-phase study shows a tendency for TOT to be efficient in reducing macroscopic inflammatory lesions in the GP and to promote healing if administered several times over a period of a few days. From the second TOT onward, in the phase-1 pilot study, reductions in macroscopic changes were consistently noticed. The same finding was particularly obvious in Horse 2 of the clinical study, which was not submitted to any other type of medical or surgical treatment; and in Horse 4, which had previously been submitted to a surgical TACE procedure but had not received any additional medical treatment. In these two horses, after only two days of TOT, the disappearance of all white mycelia and alterations in the macroscopic aspect of the other lesions were recorded.

A preliminary study of TOT showed good tolerance in standing horses kept in stocks with the head attached to both sides of the front gate [23]. The purpose of this restraint is to keep the head at shoulder level and to avoid the passive opening of the GP pharyngeal orifices. Passive opening of these orifices occurs during mastication and breathing when the horse is maintained with its head at floor level [27]. With the orifice being closed most of the time with this restraint, we hypothesized that this restraint would increase the local presence of oxygen, which could enhance the activity of defensive cells, helping to reduce the pathogenicity of the microorganisms. In this two-phase study, the GP was considered as a flexible chamber in which oxygen is delivered, at a rate of 9 to 15 L/min, via a catheter connected to a bottle of oxygen or to a wall outlet of master oxygen system, in some of the clinical cases. We decided to increase oxygen administration time and delivery rate in the clinical study based on the absence of deleterious effects of TOT at 9 L/min during the pilot study. In addition, GPM is often a subacute-to-chronic condition with necrosis of the respiratory mucosa combined with bacterial and/or mycotic infection, and protocols developed in humans to treat similar chronic wounds suggest much longer periods of oxygen therapy. Delivering oxygen for 24 h, 7 days a week was recommended [28].

In the phase-1 pilot study, the homogeneity of agreement between evaluators described by Krippendorff’s alpha coefficient was the lowest (0.476) 48 h after the first treatment (T3), which corresponds to a moderate agreement. At that time, endoscopic images only showed subtle macroscopic differences between both GPs of the same horse, which not all evaluators were able to observe. This was not the case when a GP shows no sign of inflammation before starting the study (T1), or only scarring at the end of the study (T8), in which case a perfect agreement was obtained. The results also indicate that prolonged treatment leads to scoring improvement at an earlier time point in the treated GP. Stronger statistical evidence of efficacy would probably have been reached if all eight horses of the study received four TOT administrations. However, when designing this pilot study, no information was available on the rate of oxygen administration or the time and frequency of TOT needed to obtain significant effects. This reduction in the significance of the study does not negate the finding that a trend toward a dose–effect relationship of TOT on the development of macroscopic inflammatory lesion exists with more TOT administrations producing better results.

Based on the results of the pilot study, a protocol was established for clinical cases presented at our hospital (phase-2 clinical study). As no deleterious effect of TOT at 9 L/min for 30 min was recorded, we decided to administer oxygen at 15 L/min as often as possible based on references from the human literature and for periods of 45 to 60 min depending on the horse’s tolerance. If the affected GP was not filled with blood and a TACE procedure was recommended, the first TOT session was performed during general anesthesia, as in Horses 1, 5, and 6, without disturbing the embolization procedure.

During TOT sessions in the standing position, the head was kept at shoulder height [23], and horses were not allowed to eat so as to keep the pharyngeal orifices closed as much as possible [27]. The GP catheter was changed as required, and the most common reason for changing was its displacement outside the GP caused by horses rubbing their nose and destroying the catheter fixation. For this reason, apart from meals and TOT sessions, we recommend attaching a grazing muzzle to the halter and planning to change the GP catheter once every 7 to 10 days.

Horse 2 provided important observations confirming the efficacy trend of TOT described in the pilot study. This horse presented with major bilateral GPM that did not require a TACE procedure at admission, favoring immediate TOT. Within 48 h of admission, the macroscopic size reduction and change in the appearance of the lesions definitely ruled out the need for arterial occlusion. In this particular situation, we either witnessed a spontaneous regression [2,5,9], which is unlikely, or observed a mechanism that reversed the course of the condition with progressive and complete resolution of the lesions consequently. In horses, GPM is a rarely diagnosed condition at the clinical stage, as it does not affect the vital arteries. Unfortunately, this reality does not allow creating a group of clinical cases of sufficient size only treated with TOT. Oxygen therapy is probably helping the body’s natural defenses and having a deleterious effect on the development of microorganisms present in the GP. In the case of GPM, a mixture of fungi, bacteria, and necrotic tissue coexists in the GP. In necrotizing soft tissue infections, local tissue hypoxia [29] is observed with sequestration of leukocytes, sometimes mediated by toxins such as the clostridial theta toxin [19]. The cornerstones of therapy in human patients are surgical debridement, antibiotics, and oxygen therapy. In horses, debridement or topical treatment is difficult because of the anatomical location of the GP [30,31]. However, in Horses 4 and 5, while performing URTE, we performed partial and superficial removal of mycotic plaques because major arteries were already occluded. Since oxygen has very poor solubility in liquids, removing layers of moisture should increase oxygen contact with the underlying GP mucosa, therefore providing fuel to local defenses and enabling the regeneration of cells [20]. The beneficial effects of oxygen therapy in the human literature include refractory wounds [32,33], inhibition of microorganism growth [34], increase in capillary arborization, and resistance to infection [35], as well as an effect on invasive fungal infections [36,37]. In horses, oxygenotherapy was suggested to stimulate tissue [38], and a retrospective study in seven horses reported the use of oxygen administration in the GP using a Foley catheter at 5 L/min three times daily for 20 min [22]. However, this study did not assess oxygen contribution, given the presence of various concomitant treatments such as topical agents.

Reports of spontaneous GPM regression exist [5,9], and regression was observed, at D60, in all eight nontreated GPs in this pilot study. Conversely, Horse 4 of the phase 2 study developed a right GPM several weeks after embolization of a right ICA aneurysm. The absence of ICA pulsation, after surgery and two months later, was confirmed with URTE, showing the efficacy of the TACE procedure. This indicates that arterial flow is probably not a prerequisite for providing the necessary environment for fungal colonization of a GP in horses.

After TOT administrations, combined or not with a TACE procedure, various levels of improvement in neurological disorders were observed in the following weeks. Neural insult from the fungal disease within the GP [4] induces a neuromuscular process that causes the primary functional inability of the oropharynx, resulting in dysphagia [39]. Mycotic neurotoxins probably also play a role in the inflammation of the cranial nerves [2]. In horses, it is extremely challenging to create a research model that can mimic neurological disorders related to GPM, making it difficult to validate a therapy to solve such problems. However, in this study, the total recovery of dysphagia in three horses, improvement in the condition in two others, and overall improvement or cure of ten neurological disorders out of a total of twelve is encouraging. In this small series of cases, the success rate for partially or totally solving neurological disorders (10/12) is equivalent to and even better than the 50% (9/18) success rate previously reported in a larger group of horses only treated with a TACE procedure [6]. For Horse 2 with laryngeal hemiparesis and dysphagia, improvement can be attributed to TOT only. For the other cases, the combination of TOT, and TACE is probably at the origin of improvement, which makes it possible to propose a multimodal approach of treatment for GPM. Scar tissue formation around nerves or irreversible neuronal degeneration is believed to be the cause of failed treatment to reverse neurological symptoms [4]. In the present phase-1 study, none of the scar tissue observed at D60 induced adverse neurologic disorders, suggesting that mycotic neurotoxins [40] are probably another determining factor in the pathogenesis of GPM.

In this clinical study, with the exception of hematological changes related to loss of blood in case of epistaxis, hematological and biochemical analyses did not provide any determinant element to allow a specific GPM diagnosis or treatment follow-up. On the basis of this study and our experience with more recent cases, it may be recommended to institute TOT in one or both GPs as soon as the diagnosis of GPM is made and to continue this treatment for about ten days. This clinical approach seems to reverse the course of the disease probably by affecting the development of fungi and increasing the effectiveness of the body’s natural defenses. After starting TOT, no increase in macroscopic inflammatory lesions or symptoms related to GPM was noted in the 14 horses of this two-phase study. In the case of bilateral GPM, TOT can be performed bilaterally and simultaneously such as in Horse 2 via the placement of two different catheters.

## 14. Conclusions

In the context of a GPM and based on this small number of horses, TOT can be recommended in two different clinical presentations. If no history of epistaxis exists, and if lesions are not overlying major arteries, TOT alone can be instituted. In this case, URTE is essential to assess the regression of the lesions moving away from vessels. In the case of a patient at risk of epistaxis, a multimodal approach with a TACE procedure should be performed. In both situations, TOT administration four times per day at 15 L/min for one to two weeks is probably sufficient to reverse the course of the underlying disease.

## Figures and Tables

**Table 1:** Clinical findings, treatment, and outcome for horses in the phase-2 clinical study.

Horse	History & Symptoms on Admission			Affected GP	TACE + TOT under GA (Yes/No)	TOT Standing	URTE Mycotic Lesions D2–D10	Symptoms at Discharge			Outcome (Time Post Admission)
	Nasal Discharge	Epistaxis	Neurological Disorder					Nasal Discharge	Epistaxis	Neurological Disorders	
1 FSH, 15 years Gelding, 417 kg	2 m, bilateral	-	Dysphagia g4, left-LH gIV permanent DDSP	L	L yes	L bid 5d	Melting, reduced	Resolved	-	Dysphagia g3, left-LH gIII-2 DDSP permanent	Dysphagia g2 Recover weight (15 w)
2 Thb, 4 years Mare, 426 kg	3 w, bilateral	-	Dysphagia g3, left-LH gII	L, R	-	L, R bid 27d	Melting, reduced	Resolved	-	Resolved	Won first race (24 w)
3 FSH, 5 years Gelding, 485 kg	-	Bilateral	Dysphagia g2, left-LH gII	L	L no	L bid 5d	Melting, reduced	-	Resolved	Resolved	Normal endurance activity (24 w)
4 Holstein,10 years Mare 475 kg	-	Unilateral 2 m earlier from an aneurysm of the right ICA	-	R	R (ICA 2m earlier) no	R tid 14d	Melting, reduced, debride	-	-	-	Normal sports activity (24 w)
5 FSH, 6 years, Mare 518 kg	1 w, bilateral	-	Dysphagia g4, right-LH gIV DDSP intermittent Horner syndrome	L < R	R yes	R qid 15d	Melting, reduced, debride, fistule	Reduced	-	Dysphagia g2, right-LH gIV	Nasal discharge resolved Dysphagia g1 LH gIII-2 Broodmare, pregnant (18 w)
6 Pony, 10 years Gelding 428 kg	6 w, bilateral	-	Dysphagia g2	R	R yes	R tid 9d	Melting, reduced	Resolved	-	Resolved	Normal training for Eventing (7 w)

FSH: French sports horse; Thb: thoroughbred; GP: guttural pouch; GA: general anesthesia; LH: laryngeal hemiparesis; DDSP: dorsal displacement of the soft palate; TACE: transarterial coil embolization; TOT: topical oxygen therapy; URTE: upper respiratory tract endoscopy; ICA: internal carotid artery; R/L: right/left; g: grade; w: week.

## Data Availability

Data is contained within the article or Supplementary Materials.

## Supplementary Materials

The following are available online at https://www.mdpi.com/article/10.3390/ani11113329/s1, Supplementary Item S1: Agreement between six evaluators regarding endoscopic assessment of the GP at eight different periods, Supplementary Item S2. Endoscopic assessment by six evaluators for each of the eight time periods, Supplementary Item S3. Kolmogorov–Smirnov test to evaluate the difference between treated and non-treated GPs.
