# Supplementary material for: The Effect of Topical Oxygen Therapy in Horses Affected with Mycosis of the Guttural Pouch: An Experimental Pilot Study and a Case Series
Source: Animals (Basel). 2021 Nov 22;11(11):3329. doi: 10.3390/ani11113329 (PMC8614901; doi:10.3390/ani11113329)
Supplement: Supplementary file 1 [file animals-11-03329-s001.zip › Supplementary Item 1.pdf]

Supplementary Item S1.

Agreement between six evaluators about endoscopic assessment of the guttural pouch at eight different time points.

Evaluation period	α [1]
T 1 (D-5)	1
T 2 (D0)	0,91
T 3 (D2)	0,476
T 4 (D4)	0,76
T 5 (D6)	0,817
T 6 (D8)	0,793
T 7 (D20)	0,779
T 8 (D60)	1

T = Time point of evaluation; D = day

[1]Krippendorff's alpha coefficient indicates a*:* poor (*α* <0), slight (*α*= 0 to 0.2), fair (*α*=0.21 to 0.40), moderate (*α*=0.41 to 0.60), substantial (*α*=0.61 to 0.80), and near perfect (*α*=0.81 to 1) agreement.
