# Supplementary material for: The Effect of Topical Oxygen Therapy in Horses Affected with Mycosis of the Guttural Pouch: An Experimental Pilot Study and a Case Series
Source: Animals (Basel). 2021 Nov 22;11(11):3329. doi: 10.3390/ani11113329 (PMC8614901; doi:10.3390/ani11113329)
Supplement: Supplementary file 1 [file animals-11-03329-s001.zip › Supplementary Item 2.pdf]

Supplementary Item S2.

Endoscopic assessment of macroscopic inflammatory lesions by six evaluators for each of the eight-time points.

Horse	GP	Time period (T)							
		T1	T2	T3	T4	T5	T6	T7	T8
		D-5	D0	D2	D4	D6	D8	D20	D60
1	R	0	2	5	0.9	3.2	1.5	0.5	0
	L*	0	3.7	5.4	0.9	4.4	3.6	2	0
2	R*	0	6.1	5.4	6.5	6.5	6.9	3.7	0
	L	0	4	4	4.1	4.9	4.4	1.2	0
3	R*	0	6.9	5.1	4.2	4.5	5.1	4	0
	L	0	6.9	5.5	6.1	8.1	5.5	5.5	0
4	R	0	5.3	5.5	6.6	6.6	8.1	5.9	0
	L*	0	4.6	4.4	5.4	5.4	5.2	4.7	0
5	R*	0	1	5.1	5	4.4	5.1	2.7	0
	L	0	1	5.5	6	8.2	7	4.5	0
6	R*	0	2.5	2.7	4.2	3.4	1.9	0.9	0
	L	0	1.7	4.1	7.9	7	5.6	3.6	0
7	R	0	4.5	6.2	7.6	7.9	9.4	5.6	0
	L*	0	4	5	4.7	4.1	4.9	3.2	0
8	R	0	1.1	7.1	7.1	8.6	7.6	5.5	0
	L*	0	1	5	4	5	3.6	2.4	0

D = day; R = right guttural pouch; L= left guttural pouch; (*) indicate the guttural pouch treated. Gray colour of the cells indicates for each horse the time period of treatment and subsequently indicates if the horse had 1, 2, 3 or 4 TOT.
