# Supplementary material for: The Effect of Topical Oxygen Therapy in Horses Affected with Mycosis of the Guttural Pouch: An Experimental Pilot Study and a Case Series
Source: Animals (Basel). 2021 Nov 22;11(11):3329. doi: 10.3390/ani11113329 (PMC8614901; doi:10.3390/ani11113329)

Supplementary Item S3.

Kolmogorov-Smirnov test to evaluate the difference between treated and non-treated GP.

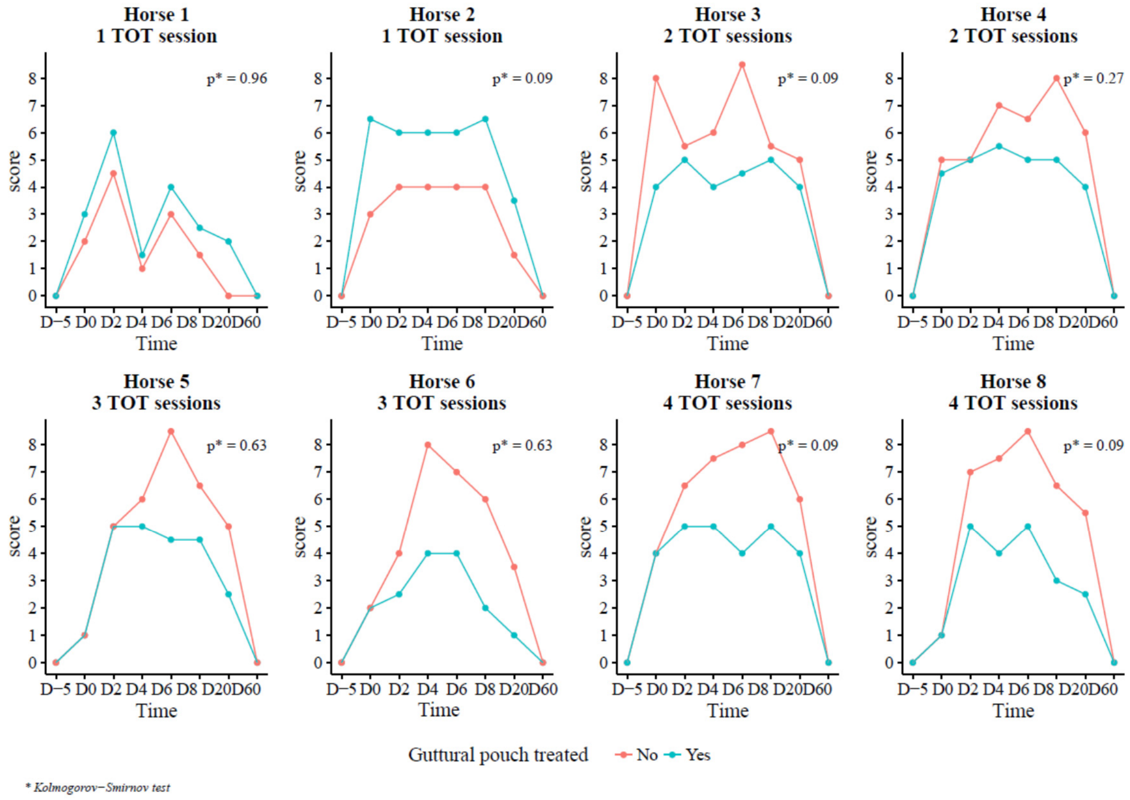

Supplement: Supplementary file 1 [file animals-11-03329-s001.zip › Supplementary Item 3.pdf]
